# Spectral tuning mediated by helix III in butterfly long wavelength-sensitive visual opsins revealed by heterologous action spectroscopy

**DOI:** 10.1186/s40851-019-0150-2

**Published:** 2019-12-16

**Authors:** Tomoka Saito, Mitsumasa Koyanagi, Tomohiro Sugihara, Takashi Nagata, Kentaro Arikawa, Akihisa Terakita

**Affiliations:** 1 Department of Biology and Geosciences, Graduate School of Science, Osaka City University, Osaka, 558-8585 Japan; 2 The Osaka City University Advanced Research Institute for Natural Science and Technology, Osaka City University, Osaka, 558-8585 Japan; 3 Laboratory of Neuroethology, SOKENDAI (The Graduate University for Advanced Studies), Shonan Village, Hayama, 240-0115 Japan

**Keywords:** Rhodopsin, Color vision, Red sensitivity, Convergent evolution

## Abstract

Absorption spectra of opsin-based pigments are tuned from the UV to the red regions by interactions of the chromophore with surrounding amino acid residues. Both vertebrates and invertebrates possess long-wavelength-sensitive (LWS) opsins, which underlie color vision involving “red” sensing. The LWS opsins have independently evolved in each lineage, which suggests the existence of diverse mechanisms in spectral tuning. In vertebrate LWS opsins, the mechanisms underlying spectral tuning have been well characterized by spectroscopic analyses with recombinant pigments of wild type (WT) and mutant opsins. However in invertebrate LWS opsins including insect ones, the mechanisms are largely unknown due to the difficulty in obtaining recombinant pigments. Here we have overcome the problem by analyzing heterologous action spectra based on light-dependent changes in the second messenger in opsin-expressing cultured cells. We found that WTs of two LWS opsins of the butterfly, *Papilio xuthus*, PxRh3 and PxRh1 have the wavelengths of the absorption maxima at around 570 nm and 540 nm, respectively. Analysis of a series of chimeric mutants showed that helix III is crucial to generating a difference of about 15 nm in the wavelength of absorption maxima of these LWS opsins. Further site-directed mutations in helix III revealed that amino acid residues at position 116 and 120 (bovine rhodopsin numbering system) are involved in the spectral tuning of PxRh1 and PxRh3, suggesting a different spectral tuning mechanism from that of primate LWS opsins.

## Background

Many animals use light information for both visual and non-visual functions. In most cases, animals capture light through an opsin, which binds to a chromophore retinal to form a light-sensitive pigment. Opsin-based pigments drive G protein-mediated signal transduction in the form of light-sensitive G protein-coupled receptors (GPCRs) [1]. Diversification of opsin protein moieties through molecular evolution has thus created diverse visual and non-visual pigments that serve as molecular bases of photoreception in various physiological phenomena.

Thousands of opsins have been identified from a wide variety of animals thus far. These are classified into at least eight groups, which diversified early in the evolution of animals [2, 3]. Each group contains opsins that form the pigments with different absorption spectra, suggesting that multiple diversification events occurred independently in each group lineage, and even in each animal lineage. Opsin diversification generally promotes the evolution of color vision, because color vision often requires spectroscopically different opsins. Basically, interaction of retinal, a chromophore, with its surrounding amino acid residues tunes the absorption spectrum of the chromophore, namely the absorption spectrum of the opsin-based pigment. Because substitutions of amino acid residues surrounding the chromophore occurred during opsin evolution, absorption spectra of opsin pigments belonging to different opsin groups may be tuned by different mechanisms.

Visual opsins of vertebrate and invertebrate (protostome) visual systems generally belong to different opsin groups; i.e., transducin (Gt)-coupled and Gq-coupled opsin groups, respectively [1, 3]. Members of these two opsin groups have an essential amino acid residue for visible light absorption at different positions, suggesting different spectral tuning mechanisms for visible light absorption. The chromophore binds to the specific lysine residue in opsins (Lys 296, according to the bovine numbering system) through Schiff base linkage. Protonation of the Schiff base is essential for visible light absorption [4–6]. However, the proton on the Schiff base is not stable inside the opsin, and it must be stabilized by a negatively charged amino acid residue called “counterion” in order to achieve visible light absorption. The counterions identified for Gt-coupled and Gq-coupled visual opsins are a glutamic acid (Glu) at 113 and 181, respectively [7–10]: the vertebrate and invertebrate visual opsins appear to employ distinct spectral tuning mechanism.

Expression of site-specific and/or chimeric mutant opsins in cultured cells and analyses of their absorption spectra have contributed to the identification of amino acid residues and/or helices involved in the spectral tuning of opsins. Such analyses have revealed that, in most vertebrate long-wavelength-sensitive (LWS) opsin-based pigments, a chloride ion binds to His181, which results in a red-shift of about 30 nm [11–13]. In invertebrate opsins, however, position 181 is occupied by glutamic acid (Glu181), which serves as a counterion, but is not involved in chloride binding. Therefore, the absorption spectra of invertebrate LWS opsin-based pigment appears to be tuned by a mechanism distinct from that of vertebrate LWS opsins.

Mechanisms other than chloride binding are also known in vertebrate opsins. Primates possess two LWS opsins, which diverged around 30 million years ago and share approximately 95% of amino acid sequence. The LWS opsin-based pigments have their absorption maxima at about 530 nm and 560 nm. Site-directed mutagenesis revealed that the difference of 30 nm is attributed to the substitution of three amino acid residues, Ala/Ser164 in helix IV and Phe/Tyr261 and Ala/Thr269 in helix VI [14–18].

In contrast to vertebrate visual opsins, spectral tuning mechanisms of invertebrate visual opsins have not been well characterized due to the difficulty in obtaining volumes of recombinant opsin-based pigments sufficient for spectroscopic analyses. A butterfly, *Papilio xuthus*, has three opsins forming the pigments sensitive to long-wavelength light. Of these three opsins, PxRh1 and PxRh3 are phylogenetically close to each other and share 310 of 379 (81.7%) amino acids [19]. The spectral sensitivity of the PxRh3-expressing photoreceptors has been electrophysiologically determined; it peaks at 600 nm with an aberrantly narrow profile compared to the predicted absorption spectrum of a visual pigment, with an absorption peak of 600 nm. This sharpening is due to the filtering effect of the red screening pigment. We have estimated the absorption spectrum of PxRh3 based on the spectral sensitivity and the screening pigment’s absorption spectrum; the PxRh3-based pigment appeared to have an absorption peak at 575 nm. Similarly, PxRh1 has been estimated to form a 545 nm visual pigment [20]. Thus, their peak absorption wavelengths are 30 nm apart, as is the case for the primate LWS opsins [21, 22]. Although we previously reported amino acid residues crucial for the spectral tuning of violet (450 nm) and blue-sensitive (420 nm) opsins in the small white butterfly, *Pieris rapae cucivora* [23], no amino acid residues involved in the spectral tuning of butterfly LWS opsins have been identified.

We recently found that heterologous action spectroscopy is useful for estimating the absorption spectrum of pigments for which it is difficult to obtain purified recombinant pigments. This method is a combination of a cAMP-dependent luciferase reporter assay with a chimeric opsin possessing the third intracellular loop of Gs-coupled jellyfish opsin [24]. In the present study, we applied this method to identify helices or amino acid residues that are crucial for spectral tuning to generate a difference of ~ 30 nm in maximum wavelength between butterfly PxRh1 and PxRh3. We also discuss the potential spectral tuning mechanism in butterfly LWS opsins.

## Results and discussion

**Fig. 1:**
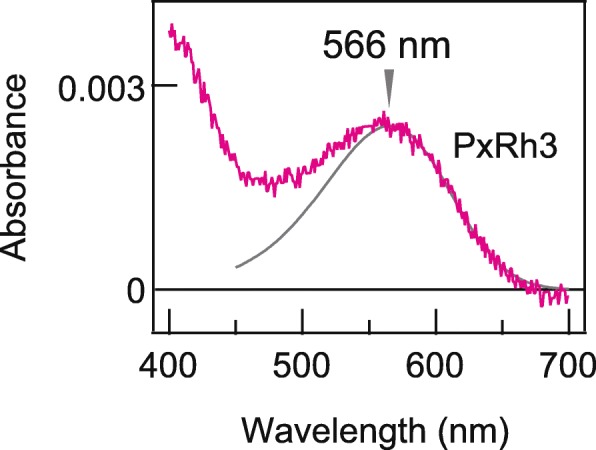
Absorption spectrum of a butterfly LWS opsin. Absorption spectra of purified PxRh3 WT (magenta). An estimated sensitivity curve of PxRh3 (grey curve) was obtained by fitting PxRh3 spectra with rhodopsin nomogram. Wavelength of maximum sensitivity of PxRh3 is estimated to 566 nm (grey arrowhead)

We expressed wild type (WT) PxRh1 and PxRh3 in cultured cells and purified the recombinant pigments to measure their absorption spectra (Fig. 1). We obtained an absorption spectrum of PxRh3, showing its absorption maximum at ~560 nm. Because scattering affects the absorption spectrum in the shorter wavelength region, especially in such a low-concentration sample, the absorption spectrum in the longer wavelength region was fitted with the rhodopsin nomogram [25] to estimate the wavelength of the absorption maximum (λ_max_). The estimated λ_max_ was 566 nm, which is ~ 10 nm shorter than previously predicted values [20], probably due to the effect of detergent [26]. Unfortunately, we did not obtain any absorption spectra for PxRh1, probably due to its very low expression level in cultured cells and/or its low stability in the detergent. We concluded that it was not possible to proceed with a comprehensive comparation of the absorption spectra of purified WT and mutant PxRh1 and PxRh3 proteins.

**Fig. 2:**
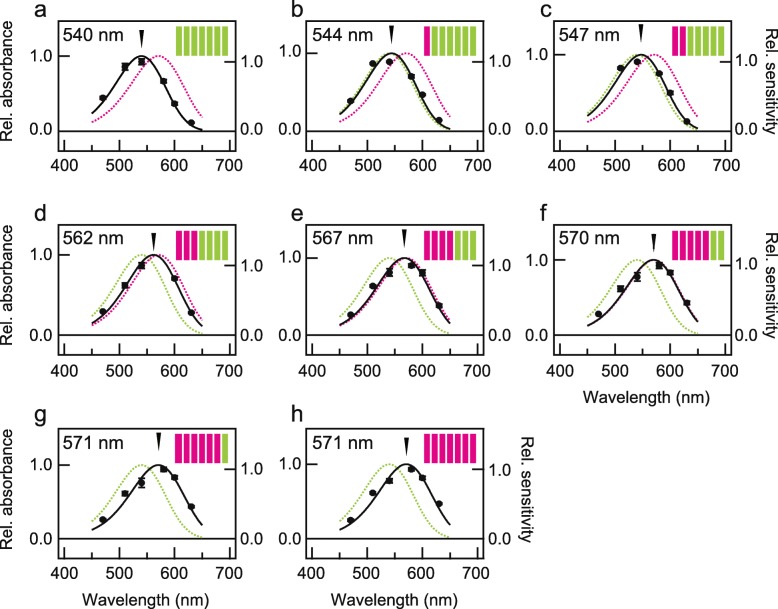
Estimated absorption spectra of chimeric mutants of PxRh1 and PxRh3. The absorption spectra of WT and chimeric mutants with respect to the transmembrane helix between PxRh1_Gs (**a**) and PxRh3_Gs (**h**), Rh3(I)/Rh1(II-VII) (**b**), Rh3(I,II)/Rh1(III-VII) (**c**), Rh3(I-III)/Rh1(IV-VII) (**d**), Rh3(I-IV)/Rh1(V-VII) (**e**), Rh3(I-V)/Rh1(VI,VII) (**f**), Rh3(I-VI)/Rh1(VII) (**g**) estimated by heterologous action spectroscopy. Solid circles represent the mean relative sensitivities of cultured cells expressing each of WT or chimeric mutant at each wavelength of light irradiation (**a** and **h**; *n* = 9, b-g; *n* = 3) and black curves indicate estimated absorption spectra. Error bar represents standard errors. The estimated absorption spectra of PxRh1_Gs and PxRh3_Gs are also indicated by green and magenta broken curves, respectively. Schematic drawings of seven transmembrane structures of butterfly opsins are also shown, in which helices of PxRh1 and PxRh3 are indicated by green and magenta, respectively

Since the *P. xuthus* opsins are Gq-coupled, we engineered Gs-coupled versions of both (PxRh1_Gs and PxRh3_Gs) by replacing their third cytoplasmic loops with that of the Gs-coupled jellyfish opsin in both WTs and mutants to enable heterologous action spectroscopy. The spectral sensitivities of PxRh1_Gs- and PxRh3_Gs-expressing cells were measured independently three times. Averaged λ_max_ values of the absorption spectra of PxRh1 and PxRh3 were estimated as 539 ± 1 nm (539 nm, 540 nm and 541 nm) and 570 ± 2 nm (569 nm, 571 nm and 572 nm), respectively, indicating that the action spectroscopy provided reproducible λ_max_ values (Additional file 1: Figure S1, see also Fig. 2a and h, which are quite close to the predicted values) [20]. These results suggest that heterologous action spectroscopy is a powerful method for investigating λ_max_ values of mutants to obtain insights into the spectral tuning mechanisms of PxRh1 and PxRh3.

In order to determine which helices of the opsins are involved in spectral tuning to generate the 30-nm difference in λ_max_, we created chimeric mutants in which one or more helices of PxRh1_Gs were replaced with the corresponding helix or helices of PxRh3_Gs (Additional file 2: Figure S2) in cultured cells. We then measured the heterologous action spectra of the recombinant cells (Fig. 2b-g). The chimeric mutant with helix I of PxRh3 and helices II-VII of PxRh1, Rh3(I)/Rh1(II-VII) had its λ_max_ at 544 nm (Fig. 2b), which is 4 nm red-shifted from that of PxRh1_Gs (referred to as Rh1 WT, Fig. 2a). Replacement of helix II in addition to helix I, designated as Rh3(I, II)/Rh1(III-VII), further caused a 3 nm red-shift of λ_max_ from Rh3(I)/Rh1(II-VII) (Fig. 2c). Interestingly, replacement of helices I-III resulted in a 15 nm red-shifted λ_max_ (562 nm), from that of Rh3(I, II)/Rh1(III-VII) (Fig. 2d). We measured the spectra of three more mutants, Rh3(I-IV)/Rh1(V-VII), Rh3(I-V)/Rh1(VI, VII) and Rh3(I-VI)/Rh1(VII), which exhibited more or less similar red-shifted λ_max_ values from Rh3(I-III)/Rh1(IV-VII), 567 nm, 570 nm and 571 nm, respectively (Fig. 2e-g). The large effect of helix III is marked, suggesting that helix III plays an important role in spectral tuning.

**Fig. 3:**
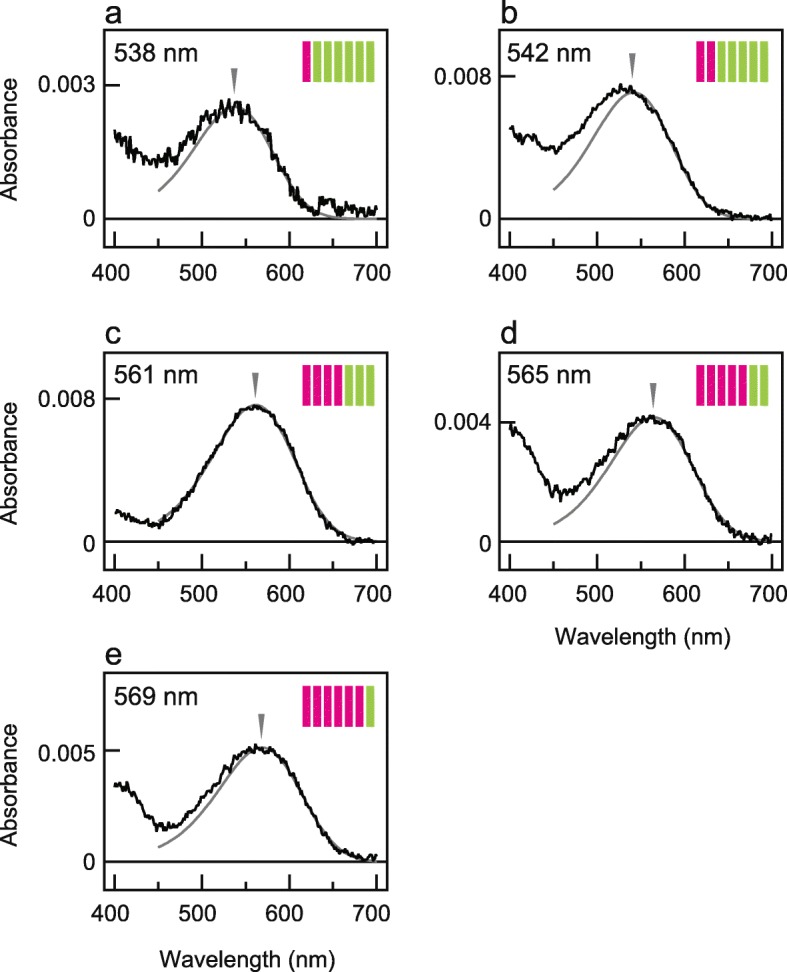
Absorption spectra of purified chimeric mutants of PxRh1 and PxRh3. Absorption spectra of purified chimeric mutants with respect to the transmembrane helix between PxRh1 and PxRh3 WTs, Rh3(I)/Rh1(II-VII) (**a**), Rh3(I,II)/Rh1(III-VII) (**b**), Rh3(I-IV)/Rh1(V-VII) (**c**), Rh3(I-V)/Rh1(VI,VII) (**d**), Rh3(I-VI)/Rh1(VII) (**e**). Estimated sensitivity curves calculated using rhodopsin nomogram (grey curve) and their absorption maxima (arrowheads) are also shown. Schematic drawings of seven transmembrane structures of butterfly opsins are also shown, in which helices of PxRh1 and PxRh3 are indicated by green and magenta, respectively

We studied absorption spectra of purified pigments in some chimeric mutants between WTs of PxRh1 and PxRh3 (NOT Gs-coupled versions, Fig. 3) to confirm the function of helix III. We first sought to measure the absorption spectrum of the purified chimeric mutant Rh3(I- III)/Rh1(IV-VII), in which helix III of PxRh3 was additionally introduced into Rh3(I, II)/Rh1(III-VII), but were unable to do so. We found a red-shift of ~ 19 nm in Rh3(I-IV)/Rh1(V-VII), in which helices III and IV of PxRh3 were introduced into Rh3(I, II)/Rh1(III-VII) (Fig. 3b, c). The 19 nm difference in λ_max_ between Rh3(I, II)/Rh1(III-VII) and Rh3(I-IV)/Rh1(V-VII) was almost identical to that observed in their spectra based on heterologous action spectroscopy (20 nm, Fig. 2c, e), supporting the importance of helix III in spectral tuning.

**Fig. 4:**
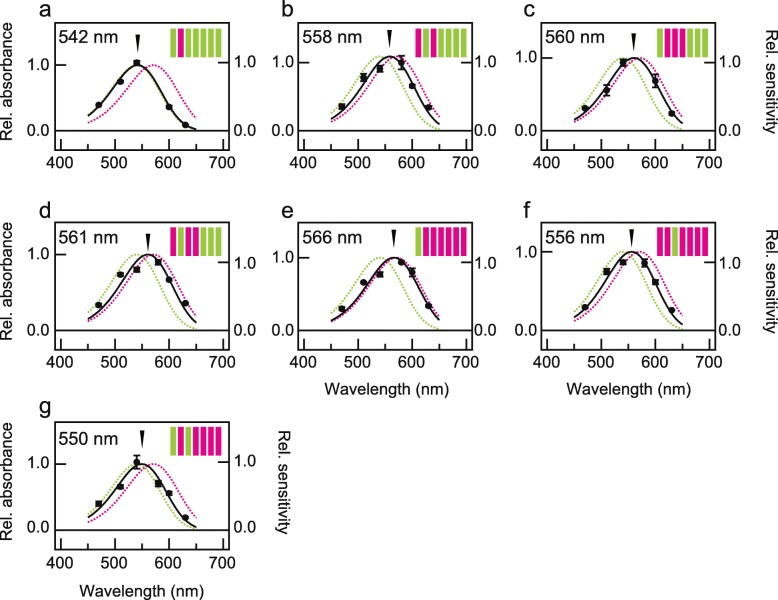
Evaluation of the involvement of helix3 of PxRh3 in the red-shift. The absorption spectra of chimeric mutants with respect to the transmembrane helix between PxRh1_Gs and PxRh3_Gs, Rh3(II)/Rh1(I,III-VII) (**a**), Rh3(I,III)/Rh1(II,IV-VII) (**b**), Rh3(II-IV)/Rh1(I,V-VII) (**c**), Rh3(I,III,IV)/Rh1(II,V-VII) (**d**), Rh1(I)/Rh3(II-VII) (**e**), Rh1(III)/Rh3(I,II,IV-VII) (**f**), Rh1(I,III)/Rh3(II,IV-VII) (**g**) were estimated by heterologous action spectroscopy. Solid circles represent the mean relative sensitivities of cultured cells expressing each chimeric mutant at each wavelength of light irradiation (*n* = 3) and black curves indicate estimated absorption spectra. The error bar shows standard error. The absorption spectra of PxRh1_Gs and PxRh3_Gs are also indicated by green and magenta broken curves, respectively. Schematic drawings of seven transmembrane structures of butterfly opsins are also shown, in which helices of PxRh1 and PxRh3 are indicated by green and magenta, respectively

Next, we investigated whether the introduction of PxRh3 helix III alone into PxRh1 could cause a 15 nm red-shift of λ_max_ by the heterologous action spectroscopy as observed between the chimeric mutants, Rh3(I, II)/Rh1(III-VII) and Rh3(I-III)/Rh1(IV-VII) (Fig. 2c, d). Unfortunately, we could not measure the spectra because the light-induced cAMP changes in cells expressing Rh3(III)/Rh1(I, II, IV-VII) were too small, probably due to low expression levels of the chimeric mutant in the cultured cells. We therefore examined other chimeric mutants with or without helix III of PxRh3 to determine the effect on the red-shift of helix III of PxRh3 (Fig. 4). Introduction of PxRh3 helix I or II alone into PxRh1 caused only a slight red-shift of λ_max_ (4 or 2 nm, respectively, Fig. 2a, b and Fig. 4a), whereas introduction of PxRh3 helix III in addition to helix I resulted in an 18-nm red-shift of λ_max_, designated as Rh3(I, III)/Rh1(II, IV-VII) (λ_max_ = 558 nm, Fig. 4b). Together with the slight red-shift observed when helix II was introduced in addition to helix I, designated as Rh3(I, II)/Rh1(III-VII) (Fig. 2c), these results suggest that the contribution of helix III is the largest of the helices tested. The λ_max_ values of mutants containing helix III together with other PxRh3 helices are consistent with this conclusion (Fig. 4c and d). This finding is also supported by the large blue-shift observed when helix III of PxRh1 was introduced to PxRh3. Introduction of the helix III of PxRh1 alone into PxRh3, designated as Rh1(III)/Rh3(I, II, IV-VII), caused a 15-nm blue-shift (λ_max_ = 556 nm, Fig. 4f). Introduction of helices I and III of PxRh1 into PxRh3, designated as Rh1(I, III)/Rh3(II, IV-VII), resulted in 21 nm blue-shift (λ_max_ = 550 nm, Fig. 4g), whereas introduction of helix I alone, designated as Rh1(I)/Rh3(II-VII), caused a blue-shift of only 5 nm (λ_max_ = 566 nm, Fig. 4e). The results suggest that helix III of both PxRh1 and PxRh3 is responsible, independent of other helices, for the spectral tuning generating the difference in λ_max_ between these proteins.

**Fig. 5:**
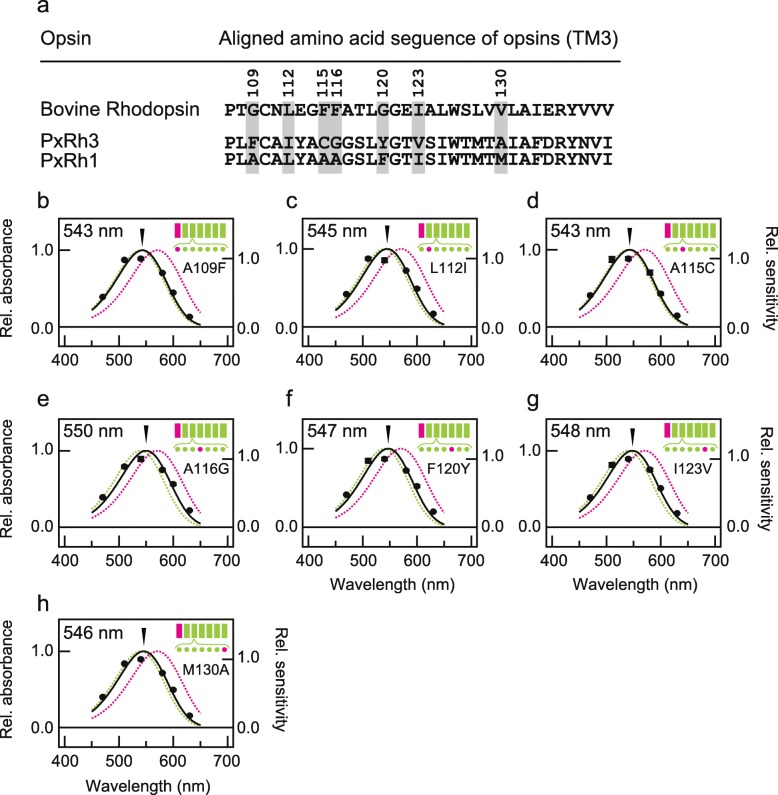
Contribution of single mutations in helix III for the red-shift in a chimera Rh3(I)/Rh1(II-VII). The absorption spectra of chimeric mutants Rh3(I)/Rh1(II-VII) having a single mutation at positions in which amino acid residues are different between PxRh1 and PxRh3in helix III (**a**, highlighted with grey boxes), Rh3(I)/Rh1(II-VII)_A109F (**b**), Rh3(I)/Rh1(II-VII)_L112I (**c**), Rh3(I)/Rh1(II-VII)_A115C (**d**), Rh3(I)/Rh1(II-VII)_A115G (**e**), Rh3(I)/Rh1(II-VII)_F120Y (**f**), Rh3(I)/Rh1(II-VII)_I123V (**g**), Rh3(I)/Rh1(II-VII)_M130A (**h**) estimated by heterologous action spectroscopy. Solid circles represent the mean relative sensitivities of cultured cells expressing each chimeric mutant at each wavelength of light irradiation (*n* = 3) and black curves indicate estimated absorption spectra. The error bar shows standard error. The absorption spectra of PxRh1_Gs and PxRh3_Gs are also indicated by green and magenta broken curves, respectively. Schematic drawings of seven transmembrane structures of butterfly opsins are also shown, in which helices and amino acid residues of PxRh1 and PxRh3 are indicated by green and magenta, respectively

**Fig. 6:**
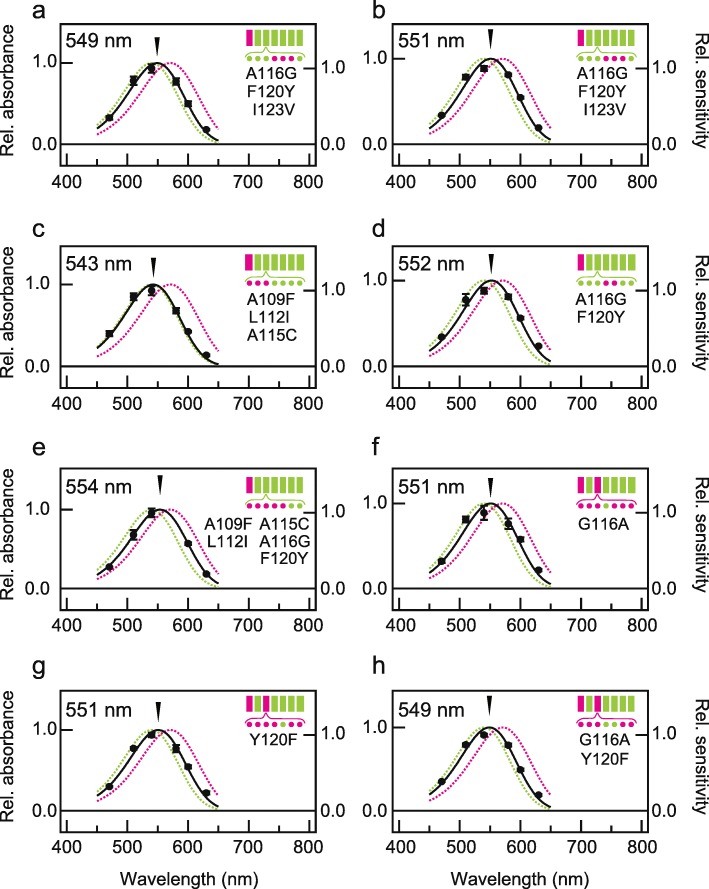
Effect of multiple mutations in helix III for the red shift in a chimera Rh3(I)/Rh1(II-VII). The absorption spectra of chimeric mutants Rh3(I)/Rh1(II-VII) having multiple mutations at positions in which amino acid residues are different between PxRh1 and PxRh3 in helix III, Rh3(I)/Rh1(II-VII)_A116G/F120Y/I123V (**a**, **b**), Rh3(I)/Rh1(II-VII)_A109F/L112I/A115C (**c**), Rh3(I)/Rh1(II-VII)_ A109F/L112I/A115C/A116G/F120Y (**d**), Rh3(I)/Rh1(II-VII)_A116G/F120Y (**e**), Rh3(I,III)/Rh1(II,IV-VII)_G116A (**f**), Rh3(I,III)/Rh1(II,IV-VII)_Y120F (**g**), Rh3(I,III)/Rh1(II,IV-VII)_G116A/Y120F (**h**) estimated by heterologous action spectroscopy. Solid circles represent the mean relative sensitivities of cultured cells expressing each chimeric mutant at each wavelength of light irradiation (*n* = 3) and black curves indicate estimated absorption spectra. The error bar shows standard error. The absorption spectra of PxRh1_Gs and PxRh3_Gs are also indicated by green and magenta broken curves, respectively. Schematic drawings of seven transmembrane structure of butterfly opsins are also shown, in which helices and amino acid residues of PxRh1 and PxRh3 are indicated by green and magenta, respectively

We investigated which amino acid residues in helix III were involved in generating different λ_max_ values between PxRh1 and PxRh3. Seven amino acid residues in helix III differ between PxRh1 and PxRh3 (Fig. 5a). We individually substituted each of these seven residues in the chimeric mutant Rh3(I)/Rh1(II-VII) (λ_max_ = 544 nm, Fig. 2b) with the corresponding amino acid of PxRh3 to test whether the additional site-specific mutation in helix III of Rh3(I)/Rh1(II-VII) shifts its λ_max_ toward that of Rh3(I, III)/Rh1(II, IV-VII) (λ_max_ = 558 nm, Fig. 4b). Single-amino-acid substitutions A116G, F120Y and I123V mutants only caused slight red-shifts, + 6 nm, + 3 nm and + 4 nm respectively (Fig. 5e-g), which were not comparable to that caused by introduction of helix III to Rh3(I)/Rh1(II-VII) (+ 14 nm, Figs. 2b and 4b). We tested triple mutations, A116G/F120Y/I123V into Rh3(I)/Rh1(II-VII) twice, but both independent measurements indicated only + 5 nm shift (Fig. 6a, b). We made three more mutants in which two, three, or five amino acids in helix III of Rh3(I)/Rh1(II-VII) were substituted (A109F/L112I/A115C and/or A116G/F120Y). The quintuple mutations, A109F/L112I/A115C/A116G/ F120Y, caused a 10-nm red-shift (λ_max_ = 554 nm, Fig. 6e). The triple mutations A109F/L112I/A115C caused a 1-nm blue-shift (λ_max_ = 543 nm, Fig. 6c), but the double mutations A116G/F120Y caused an 8-nm red-shift (λ_max_ = 552 nm, Fig. 6d). Apparently, the amino acid residues at positions 116 and 120 account for more than half of the red-shift caused by helix III in Rh3(I, III)/Rh1(II, IV-VII). To determine whether both residues are indeed involved in the red-shift caused by introduction of helix III of Rh3 to Rh3(I)/Rh1 (II-VII), we analyzed λ_max_ values of Rh3(I, III)/Rh1(II, IV-VII) mutants having reverse mutations at positions 116 and/or 120 (G116A and/or Y120F) in helix III of Rh3. Two single mutants, G116A or Y120F, were both associated with a 7-nm blue-shift (λ_max_ = 551 nm, Fig. 6f, g) and the G116A/Y120F double-mutant showed as 9-nm blue-shift (λ_max_ = 549 nm, Fig. 6h) from that of Rh3(I, III)/Rh1(II, IV-VII) (λ_max_ = 558 nm, Fig. 4b). The effect of the double mutations (9 nm) was equivalent to nearly two-thirds of the difference in λ_max_ between Rh3(I)/Rh1(II-VII) and Rh3(I, III)/Rh1(II, IV-VII), supporting that amino acid residues at positions 116 and 120 in helix III play a crucial role in spectral tuning of PxRh1 and PxRh3.

Previous studies in the primate LWSs peaking at 530 nm and 560 nm have reported that three amino acid residues in helices IV and VI are the main contributors to the generation of the 30-nm difference in their λ_max_ [14, 16–18]. We found that helix III is crucial for the spectral tuning generating the 30-nm difference in LWS opsins in butterfly. Two amino acid residues in helix III cooperatively serve as major contributors to this shift, suggesting that primate and butterfly LWS opsins employ different mechanisms to achieve similar amounts of λ_max_ shifts. Vertebrate LWS opsins, including the primate LWSs, have Glu113 as the counterion [7–9], which is different from the counterion in invertebrate opsins, Glu181 [10, 27]. Additionally, in the vertebrate LWS opsin, site 181 is occupied by His, in place of Glu, to bind to chloride ion, which shifts the absorption maximum towards red [11, 12]. This difference in counterion position appears to be related to the fact that different helices are involved in the spectral tuning in vertebrate and invertebrate LWS opsins. Interestingly, a previous study by our group suggested that displacement of the counterion from Glu181 to Glu113 during the molecular evolution of vertebrate opsins enabled a unique mutation from Glu to His at position 181 to acquire chloride ion-biding ability of the vertebrate LWS opsins [27]. The present finding on invertebrate LWS opsins retaining Glu 181 accordingly implies a more general mechanism underlying spectral tuning in LWS opsins.

A previous mutational study [16] and the crystal structure of bovine rhodopsin (1 U19) [28] suggested that interactions of the hydroxyl-bearing three amino acids in helices IV and VI with the chromophore tune the absorption spectrum of primate LWS opsins. In the crystal structure of the jumping spider rhodopsin-1 (6I9K) [29], which is a Gq-coupled visual opsin similar to PxRh1 and PxRh3, the alpha carbons of all seven amino acid residues that differ between the PxRh1 and PxRh3 helix IIIs, including those at positions 116 and 120, appear to be located on the side of helix III distal from the chromophore retinal (Additional file 3: Figure S3). This suggests a unique indirect interaction with retinal through other amino acid residue(s). In this context, because a Gly residue generally inhibits precise helix formation, Gly at position 116 in PxRh3 may destabilize the helix III to weaken direct interactions between some amino acids and retinal, causing the red-shift.

Here, we investigated the molecular mechanism underlying the shift of absorption spectra of PxRh1and PxRh3 of a butterfly *Papilio xuthus*. Purification with a detergent was not suitable to obtain absorption spectra of PxRh1 WT and some chimeric mutants between PxRh1 and PxRh3, but we were able to estimate their absorption spectra by heterologous action spectroscopy [24]. Clearly, heterologous action spectroscopy represents a potentially powerful tool for the investigation of spectral tuning mechanisms in addition to analyses of absorption characteristics of novel opsins [24] and counterions [30]. Several other Papilionid species also possess Rh1 and Rh3 [31]. Amino acid sequences of helices III in these species are identical to those in *P. xuthus* Rh1 and Rh3, respectively, suggesting that the spectral tuning mechanism found in PxRh1 and PxRh3 is conserved among Papilionid Rh1 and Rh3. In contrast, *Apodemia mormo* possess two kinds of LWS opsins, LWRh1 and LWRh2, which diverged independently of PxRh1 and PxRh3 [32]. In addition, it has been suggested that other invertebrates, such as dragonfly and mantis shrimp, have multiple LWS opsins [33, 34]. It would be of particular interest to compare spectral tuning mechanisms of independently evolved invertebrate LWS opsins by heterologous action spectroscopy.

## Conclusions

In this report, we estimate the absorption spectra of wild type and mutants of two LWS opsins, PxRh3 and PxRh1, in the butterfly *Papilio xuthus* using heterologous action spectroscopy, a method recently developed by our group. We found that two amino acids at positions 116 and 120 in helix III are crucial for the spectral tuning of butterfly LWS opsins by analyses of a series of chimeric and site-directed mutants. Since the spectral tuning sites were different from those of vertebrate LWS opsins, these findings suggest a new spectral tuning mechanism for LWS opsins. Taken together with our previous report that invertebrate opsins retain an ancestral molecular architecture, the spectral tuning mechanism of butterfly LWS opsins described here may reflect a more general spectral tuning mechanism for LWS opsins as well.

## Materials and methods

### Construction of expression vectors of PxRh1 and PxRh3 and their mutants

The cDNA of full-length PxRh1 and PxRh3 were synthesized to optimize for expression in human cells based on their amino acid sequences and tagged with the monoclonal antibody Rho 1D4 epitope sequence (ETSQVAPA) [35]. Chimeric mutants having the third intracellular loop of Gs-coupled jellyfish opsins, deduced from a previous report [36], were generated by replacing the cDNA region corresponding to the third intracellular loop of opsins with that of Gs-coupled jellyfish opsin by PCR. Chimeric mutants with respect to the transmembrane helix between PxRh1 and PxRh3 were generated by combining two fragments using PCR with primers at the ends of the combined sequence. Boundaries of helices were indicated in Additional file 2: Figure S2. Point mutations were introduced into the DNA by PCR with mutation-containing primers. The cDNAs were inserted between the Hind III and Eco RI sites of the pcDNA3.1 expression vector (Invitrogen).

### Expression and purification of opsin-based pigments and spectroscopy

Opsin expression and purification were performed as described previously [37]. Briefly, opsin expression vectors were transfected into HEK293S cells using the calcium-phosphate method. Transfected cells were harvested two days after the transfection. To reconstitute the pigment, the expressed proteins were incubated with excess amount of 11-*cis* retinal overnight. Pigments were then extracted with 1% dodecyl β-D-maltoside (DM) in HEPES buffer (pH 6.5) containing 140 mM NaCl and 3 mM MgCl_2_, bound to 1D4-agarose, washed with 0.02% DM in the HEPES buffer and eluted with the HEPES buffer containing 0.02% DM and 1D4 peptide. The absorption spectra of the opsin-based pigments were recorded at 4 °C using a Shimadzu UV2450 spectrophotometer.

### Heterologous action spectroscopy

Heterologous action spectroscopy based on changes in the intracellular cAMP level of opsin-expressing HEK293S cells was performed using the GloSensor cAMP assay (Promega), as described previously (Sugihara et al., 2016). Briefly, the opsin expression vectors were transfected into HEK293S with the pGloSensor-22F cAMP plasmid (Promega) using the PEI transfection method. The transfected cells were incubated overnight at 37 °C and after supplementation of 11-*cis* retinal, cells were incubated overnight at 25 °C. Before measurements, the culture medium was replaced with a CO_2_-independent medium containing 10% FBS and GloSensor cAMP Reagent stock solution (Promega). Luminescence derived from Glosensor, an indicator of intracellular cAMP, was measured at 25 °C using a GloMax 20/20n Luminometer (Promega). The light-induced changes in luminescence were measured by irradiation with light-emitting diode (LED) light for 5 s and the measured luminescence values were normalized to those just before the irradiations. LEDs with spectral emission peaks of 470 nm, 510 nm, 540 nm 580 nm, 600 nm and 630 nm arrayed on a board (SPL-25-CC; REVOX Inc., Kanagawa, Japan) were used as light sources for measurements of wavelength-dependent responses of opsin-expressing cultured cells. The quantum flux of each LED light was adjusted to 6.2 *10^14^ or 2.2 *10^14^ photons/cm^2^/sec using interference filters (MZ0470, MZ0510, MZ0540, MZ0580, MZ0600 and MZ0630; Asahi Spectra Co., Ltd.), neutral-density (ND) filters (SIGMAKOKI Co., Ltd., Saitama, Japan and Shibuya Optical Co., Ltd., Saitama, Japan) and ground-glass (Shibuya Optical Co., Ltd.). Dose (intensity)-response curves were generated for cultured cells expressing each of the opsins by irradiating cells with green (500 nm) LED light (Ex-DHC; Bio Tools Inc. Gunma, Japan) or orange (600 nm) LED light at multiple intensities, established using a series of neutral-density (ND) filters. It should be noted that individual dishes of cells were irradiated only once during the measurements, and at least three independent measurements were made at each wavelength or intensity. The intensity–response curve was obtained by fitting a sigmoid function (*V = V*_max_*I^*n*^ */ (I*^*n*^ *+ K*^*n*^), where *V* is the response amplitude, *V*_*max*_ is maximum response amplitude, *I* is the stimulus light intensity, *K* is stimulus intensity eliciting 50% *V*_*max*_, and *n* is the exponent) to the mean responses at each intensity of light irradiation. The amplitude of the wavelength-dependent responses were extrapolated to the intensity-response curve to transform the amplitude into photon numbers required for the responses, equivalent to the relative sensitivity [21]. Absorption spectra were estimated by fitting a rhodopsin template [25] to the relative sensitivities according to the least squares method with the aid of IGOR Pro software (WaveMetrics).

## Supplementary information

**Additional file 1: Figure S1.** The estimated absorption spectra of PxRh1_Gs and PxRh3_Gs. The absorption spectra of PxRh1_Gs (a-c) and PxRh3_Gs (d-f) estimated by heterologous action spectroscopy. Results from three independent experiments are shown. Solid circles represent the mean relative sensitivities (change in luminescence/cAMP) of cultured cells expressing PxRh1_Gs or PxRh3_Gs at each wavelength of light irradiation (*n* = 3) and curves indicate estimated absorption spectra. The error bar shows standard error.

**Additional file 2: Figure S2.** Secondary structure of PxRh3. Solid black circles indicate amino acid residues different from those of PxRh1. The third intracellular loop of PxRh3, which was replaced with that of Gs-coupled jellyfish opsin for the cAMP-based heterologous action spectroscopy, is boxed with a dotted line. Boundaries of helices for making chimeric mutants between PxRh1 and PxRh3 are indicated by black lines.

**Additional file 3: Figure S3.** Locations of amino acid residues different between PxRh1 and PxRh3 in helix III. Seven amino acid residues that are different between PxRh1 and PxRh3 in helix III are marked (red) on the tertiary structure of jumping spider rhodopsin-1 (PDB num: 6I9K) based on the alignment of butterfly opsins and jumping spider rhodopsin-1. The retinal chromophore is displayed in yellow.

## Data Availability

The datasets supporting the conclusions of this article are included within the article.
